# Benchmarking of ATAC Sequencing Data From BGI’s Low-Cost DNBSEQ-G400 Instrument for Identification of Open and Occupied Chromatin Regions

**DOI:** 10.3389/fmolb.2022.900323

**Published:** 2022-07-07

**Authors:** Marina Naval-Sanchez, Nikita Deshpande, Minh Tran, Jingyu Zhang, Majid Alhomrani, Walaa Alsanie, Quan Nguyen, Christian M. Nefzger

**Affiliations:** ^1^ Institute for Molecular Bioscience, University of Queensland, St Lucia, QLD, Australia; ^2^ Department of Clinical Laboratories Sciences, Faculty of Applied Medical Sciences, Taif University, Taif, Saudi Arabia; ^3^ Centre of Biomedical Sciences Research (CBSR), Deanship of Scientific Research, Taif University, Taif, Saudi Arabia

**Keywords:** ATAC-seq, sequencing platform, benchmarking, BGI, DNBSEQ-G400, Illumina, motif enrichment, foot-printing

## Abstract

**Background:** Chromatin falls into one of two major subtypes: closed heterochromatin and euchromatin which is accessible, transcriptionally active, and occupied by transcription factors (TFs). The most widely used approach to interrogate differences in the chromatin state landscape is the Assay for Transposase-Accessible Chromatin using sequencing (ATAC-seq). While library generation is relatively inexpensive, sequencing depth requirements can make this assay cost-prohibitive for some laboratories.

**Findings:** Here, we benchmark data from Beijing Genomics Institute’s (BGI) DNBSEQ-G400 low-cost sequencer against data from a standard Illumina instrument (HiSeqX10). For comparisons, the same bulk ATAC-seq libraries generated from pluripotent stem cells (PSCs) and fibroblasts were sequenced on both platforms. Both instruments generate sequencing reads with comparable mapping rates and genomic context. However, DNBSEQ-G400 data contained a significantly higher number of small, sub-nucleosomal reads (>30% increase) and a reduced number of bi-nucleosomal reads (>75% decrease), which resulted in narrower peak bases and improved peak calling, enabling the identification of 4% more differentially accessible regions between PSCs and fibroblasts. The ability to identify master TFs that underpin the PSC state relative to fibroblasts (via HOMER, HINT-ATAC, TOBIAS), namely, foot-printing capacity, were highly similar between data generated on both platforms. Integrative analysis with transcriptional data equally enabled direct recovery of three published 3-factor combinations that have been shown to induce pluripotency.

**Conclusion:** Other than a small increase in peak calling sensitivity for DNBSEQ-G400 data (BGI), both platforms enable comparable levels of open chromatin identification for ATAC-seq library sequencing, yielding similar analytical outcomes, albeit at low-data generation costs in the case of the BGI instrument.

## Introduction

The Assay for Transposase-Accessible Chromatin using sequencing (ATAC-seq) is the most extensively used method to identify accessible chromatin regions, on a genome-wide level. ATAC-seq identifies accessible DNA regions by probing open chromatin with a hyperactive Tn5 transposase, which inserts sequencing adapters into open regions of the genome (Buenrostro et al., 2013; Corces et al., 2017). However, transcription factors (TFs) bound to otherwise nucleosome-free DNA prevent the enzyme from cleaving, leaving small regions, referred to as TF footprints, where sequencing read depth suddenly drops within regions of high coverage (Corces et al., 2017). Accordingly, ATAC-seq data can identify regions of increased accessibility and map bound TFs that can be inferred via motif prediction (Heinz et al., 2010; Janky et al., 2014; Naval-Sánchez et al., 2015; Li et al., 2019; Bentsen et al., 2020). Differentially accessible regions between two cell states, therefore, provide information about the TFs that underlie differences in cell identity. Complementary RNA-seq data can confirm which of these TFs are expressed and give information about gene regulatory networks under their control. In addition, functional regulatory elements that underpin specific cell states, namely, promoters, enhancers, and insulators, lie within accessible chromatin and their identification is also key to unraveling gene regulatory programs governing cell identity across development and disease (Minnoye et al., 2021).

Following the technique’s initial development in 2013, an improved version of the assay was established in 2017. The so-called OMNI-ATAC-seq workflow (Corces et al., 2017) generates chromatin state data with a high percentage of usable reads and low levels of mitochondrial DNA contamination (<5% of reads compared to ∼50% for the original workflow). Unlike the standard ATAC-seq protocol (Buenrostro et al., 2013), OMNI ATAC-seq (Corces et al., 2017) has also substantially improved signal-to-background ratio and the information content (∼4-fold more unique fragments mapping to peaks), critical advantages for the identification of TF footprints.

To date, most ATAC-seq and OMNI-ATAC-seq analyses have been performed almost exclusively using Illumina sequencing platforms and it is unknown whether assay results are sequencer dependent. The Beijing Genomics Institute (BGI) is offering alternative sequencing solutions, namely, their proprietary DNBSEQ platforms, that enable sequencing at drastically reduced costs compared to Illumina platforms. The technology underlying the BGI platforms combines DNA nanoball (DNB) nanoarrays with polymerase-based stepwise sequencing (DNBseq) (Senabouth et al., 2020). Here, we benchmark BGI’s DNBSEQ-G400 instrument against Illumina’s HiSeqX10 platform for performance in the sequencing of ATAC-seq libraries. Approximate costs for generating 600–800 million reads (=300–400 million read pairs) at 100 bp with the DNBSEQ-G400 instrument are $650 USD when performed as a sequencing service at BGI. Conversion of Illumina libraries into nanoball libraries is provided as a free-of-charge service. Conversely, approximate costs for generating 600–800 million reads (=300–400 million read pairs) at 150 bp via Illumina’s HiSeqX10 are $1600 USD. While BGI platforms have been evaluated to be comparative in performance to Illumina platforms for sequencing of RNA-seq libraries (Fehlmann et al., 2016; Zhu et al., 2018; Natarajan et al., 2019; Senabouth et al., 2020) and whole-genome DNA libraries (Mak et al., 2017; Kim et al., 2021; Zhu et al., 2021), to date no study has compared Illumina and BGI platforms for their ability to sequence ATAC-seq libraries and recover the accessible cellular chromatin landscape.

For benchmarking, we used bulk OMNI-ATAC libraries from mouse Embryonic Stem Cells (mESC) and Mouse Embryonic Fibroblasts (MEFs), with their well-characterized molecular differences. We compared sequencing quality metrics, a number of regions identified as accessible per cell type, differential peak calling, and importantly, the ability to identify known master TFs driving PSC cell-type identity.

Our data show that BGI’s DNBSEQ-G400 instrument is a good option to sequence ATAC-seq libraries with overall similar outcome and accuracy (with a trend toward improved sensitivity for BGI data relative to Illumina’s HiSeqX10 platform), albeit at a lower cost.

## Results

**FIGURE 1:**
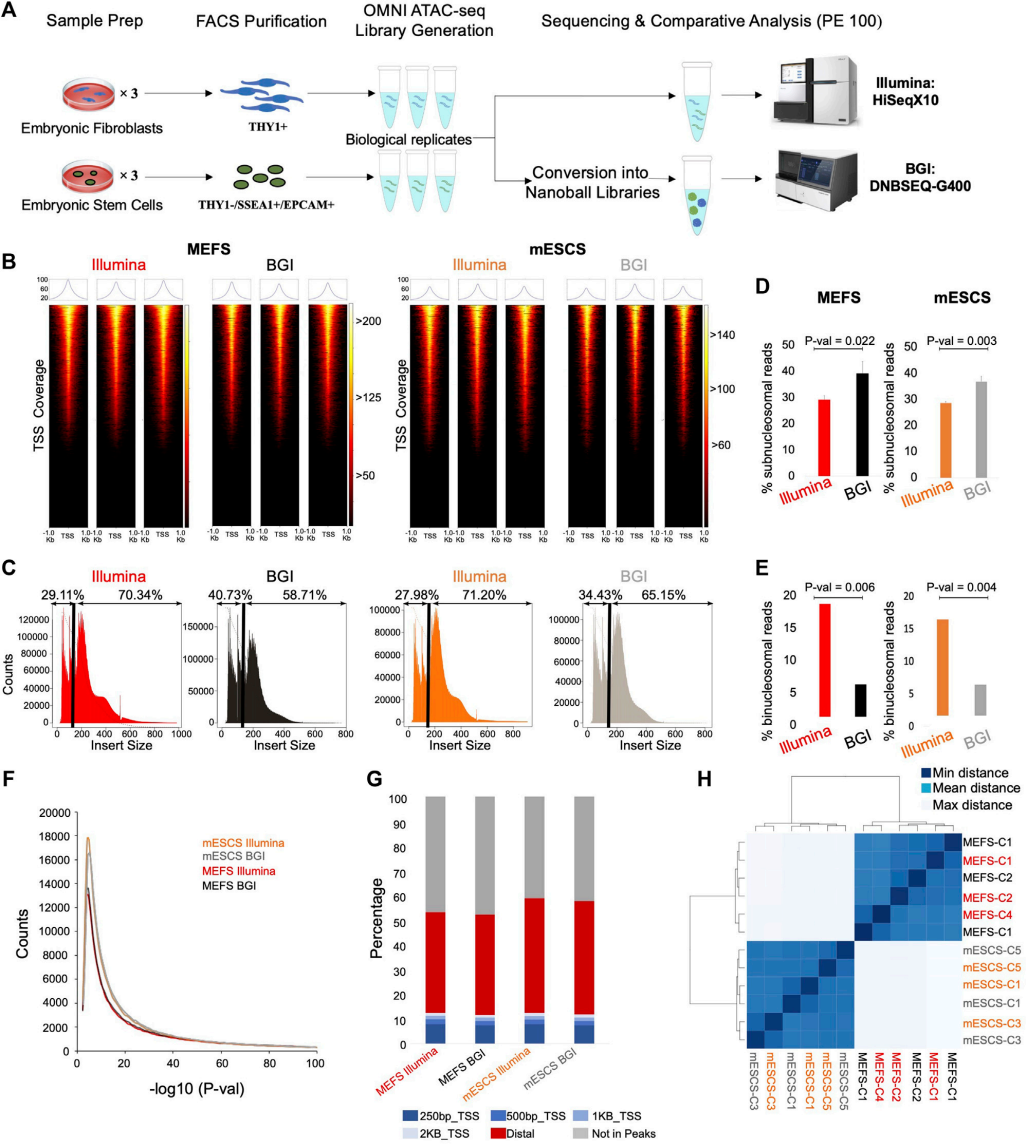
Genomic context and insert size distribution of sequenced reads. **(A)** Schematic overview of experimental setup (e.g., Omni-ATACseq library generation from three biological replicate samples for MEFs and mESCs each). **(B)** Enrichment of sequencing fragments at transcriptional start sites (TSS, ±1 Kb) for data from both platforms (*n* = 3, biological replicates). The color scale indicates the number of reads mapping to each TSS across the genome. **(C)** Representative plots depicting insert size distribution for data from the different sequencing platforms; a black line indicates the boundary between sub- and mono/poly-nucleosomal reads with relative % values indicated above. **(D)** Quantification of percentage of bi-nucleosomal reads in Illumina versus BGI data for MEFs and mESCs (*n* = 3, biological replicates, Student’s *t*-test, two-tailed, unpaired). **(E)** Quantification of the percentage of sub-nucleosomal reads in Illumina versus BGI data for MEFs and mESCs (*n* = 3, biological replicates, Student’s *t*-test, two-tailed, unpaired). **(F)** Peak calling was performed (MACS2), followed by visualization of peak numbers (averaged across the three biological replicates) with their associated *p*-values. **(G)** Genomic context of reads from each sample group (averaged across the three biological replicates). **(H)** Unsupervised clustering of all samples based on Euclidean distance on the number of counts within open-regions (*n* = 3 biological replicates) minimal distance = 0, mean distance = 315, and maximal distance = 540.

To compare the capabilities of BGI’s DNBSEQ-G400 instrument with Illumina’s HiSeqX10 platform to generate OMNI-ATAC-seq data, we used six versus six sequencing libraries from MEFs and mESCs as our case study (Figure 1A). We chose these two cell identities for benchmarking because differences between them have been well studied following Yamaka’s seminal discovery of induced pluripotent stem cells (iPSC) (Takahashi and Yamanaka, 2006), with most possible TFs that can enable pluripotency induction and/or underpin the pluripotent state relative to fibroblasts arguably now known.

To obtain cells of the highest purity as input for the OMNI-ATAC-seq assay, we performed fluorescence-activated cell sorting (FACS), to isolated THY1+ cells from fibroblast cultures and THY1-/SSEA1+/EPCAM+ cells from mESC cultures [cell surface marker profiles commonly used for their purification (Nefzger et al., 2014; Firas et al., 2014)]. OMNI-ATAC-seq libraries were generated for both cell types from three biological replicate samples each (Figure 1A). Half of the DNA from the resulting six sequencing libraries (3x MEFs, 3x mESCs) were used for sequencing on Illumina’s HiSeqX10 instrument. The remainder of the same six sequencing libraries were converted to nanoball libraries to enable sequencing on BGI’s DNBSEQ-G400 platform (Figure 1A). Accordingly, our study is directly comparing sequencing outcomes for libraries stemming from the same six OMNI-ATAC-seq reactions on different platforms. For data generation, one lane yielding 600–800 million reads (300–400 million read pairs) was used for each technology (Figure 1A).

### Comparable Quality Metrics for Both Platforms but Differences in Insert Fragment Size

The total number of reads generated for the six libraries (for three MEF and three mESC samples) ranged from 98 to 124 million reads and from 91 to 164 million reads for the Illumina and BGI sequencing platforms, respectively (Table 1; Supplementary Table 1). Considering that the libraries were sequenced on the DNBSEQ-G400 in paired-end 100 bp mode and on the HiSeqX10 in paired-end 150 bp mode, the HiSeqX10 data was down-sampled to 100 bp for best comparison. Mapping and library quality metrics, namely, the percentage of initially mapped reads, percentage of PCR duplicates, and percentage of mitochondrial DNA, resulted in highly similar numbers between both sequencing platforms (Table 1; Supplementary Table 1). Thus, the nanoball library conversion step required for sequencing the Illumina libraries on the BGI instrument did not considerably affect the proportion of PCR duplicates in the data. The final number of usable reads with a quality score greater than 10 (Q10), corresponding to a base call accuracy of 90%, yielded 70 million reads on average for the MEF libraries for both sequencing platforms (64–76 million) and of 57 million (47–65 million) and 52 million (49–56 million) reads on average for the mESC libraries for the BGI and Illumina platform, respectively (Table 1; Supplementary Table 1). To remove the potential impact of sequencing depth on the comparative analyses, however minor, we randomly down-sampled all datasets (irrespective of sequencing platform) to 60 million reads for MEFs and 45 million reads for mESCs before further processing.

**TABLE 1:** Averaged mapping statistics and quality metrics from the analysis of MEF and mESC libraries sequenced on Illumina’s HiSeqX10 and BGI’s DNBSEQ-G400 instrument. Metrics include a percentage of starting reads that mapped against the mouse reference genome, the percentage of the starting reads with identical sequence considered to be PCR duplicates, the percentage of starting reads that mapped uniquely against the reference genome, the percentage of sequencing reads that mapped against the mitochondrial genome, and the percentage of reads that were usable, defined as uniquely mapped against genomic reference genome, deduplicated and not falling in the mitochondrial genome.

Sequencing platform	Cell type	Initial number of reads	% Mapped	% Duplicates	% Uniquely mapped	% Mitochondrial reads	% Usable reads
BGI	MEFS	105,689,537	96.42	24.11	67.76	0.57	67.20
Illumina	MEFS	108,340,454	96.90	26.92	65.93	0.53	65.40
BGI	mESCS	129,503,922	97.28	45.09	46.60	0.95	45.65
Illumina	mESCS	109,009,327	97.15	41.48	50.13	1.09	49.04

ATAC-seq libraries capture accessible chromatin, which is enriched for active regulatory regions, namely, promoters. Therefore, Transcription Start Site (TSS) enrichment is used to assess a signal-to-background ratio. All six libraries present a clear and comparable overrepresentation of reads specific to TSS regions (Figure 1B; Supplementary Table 1).

The insert size distribution shows the DNA fragment length recovered through sequencing. Considering that Tn5 transposases bind to accessible DNA, this produces a downward ladder profile with nucleosome-free/sub-nucleosomal regions (<147 bp), followed by mono-nucleosomal (180–247 bp) and di-nucleosomal regions (315–473 bp) (Buenrostro et al., 2013). Comparative analysis of insert size distributions between BGI and Illumina sequencing platforms shows a larger insert size in Illumina data. When plotting the insert size distribution of the recovered sequencing fragments, an apparent increase in shorter fragments and a decrease in the proportion of larger fragments can be observed for BGI-derived data (Figure 1C). Quantification of this insert size bias for biological replicates of mESCs and MEFs showed that collectively Illumina-derived data contains significantly more di-nucleosomal reads (on average 2.6-fold higher proportion of bi-nucleosomal reads across both cell types relative to BGI data) (Figure 1D; Supplementary Table 2). Conversely BGI data recovered significantly more nucleosome-free reads (on average 1.3-fold more sub-nucleosomal reads relative to Illumina data) (Figure 1E; Supplementary Table 2). This shows a fragment size bias of BGI-derived data relative to the Illumina instrument.

To verify that truncation of the Illumina data from paired-end 150 bp–100 bp was not responsible for the observed insert size bias, we also compared BGI-derived data at 100 bp paired-end with the untruncated Illumina data at 150 bp paired-end. Our results demonstrate that truncation of Illumina data had a negligible effect on reads mapping quality (Supplementary Table 1). Likewise, the insert size bias between Illumina and BGI-derived data could be observed (Supplementary Figures 1A,B; Supplementary Table 2), demonstrating that truncation of the Illumina reads did not affect key data metrics. Therefore, all additional analyses in this study were performed with the down-scaled Illumina data at 100 bp paired-end.

### Identification of More Peaks in BGI-Derived Data due to Narrower Peak Bases

To evaluate the impact of sequencing platforms on ATAC-seq datasets, we performed peak calling for each sample. For both mESCs and fibroblasts, BGI-derived data identified as many or more peaks relative to Illumina-derived data (Figure 1F). As expected for OMNI-ATAC-seq data >50% of all mapped reads fell within TSS or distal peaks (Figure 1G) at virtually identical levels for data derived from either platform (Figure 1G; Supplementary Table 1). Mapping rates to the TSS were comparable across 250 bp, 500 bp, and 1 kb windows between Illumina and BGI-derived data (Figure 1G). Unsupervised clustering of the data (based on a merged peak set from all samples) showed cell-type-specific clustering for mESCs and fibroblasts but did not reveal subgroups based on sequencing technology, indicating an overall highly similar data structure (Figure 1H). Indeed, each of the 2 × 3 MEF and 2 × 3 mESC samples clustered closest to their respective biological replicate, indicating that the differences introduced by the two sequencing platforms were more subtle than the differences between the biological replicates themselves (Figure 1H).

**FIGURE 2:**
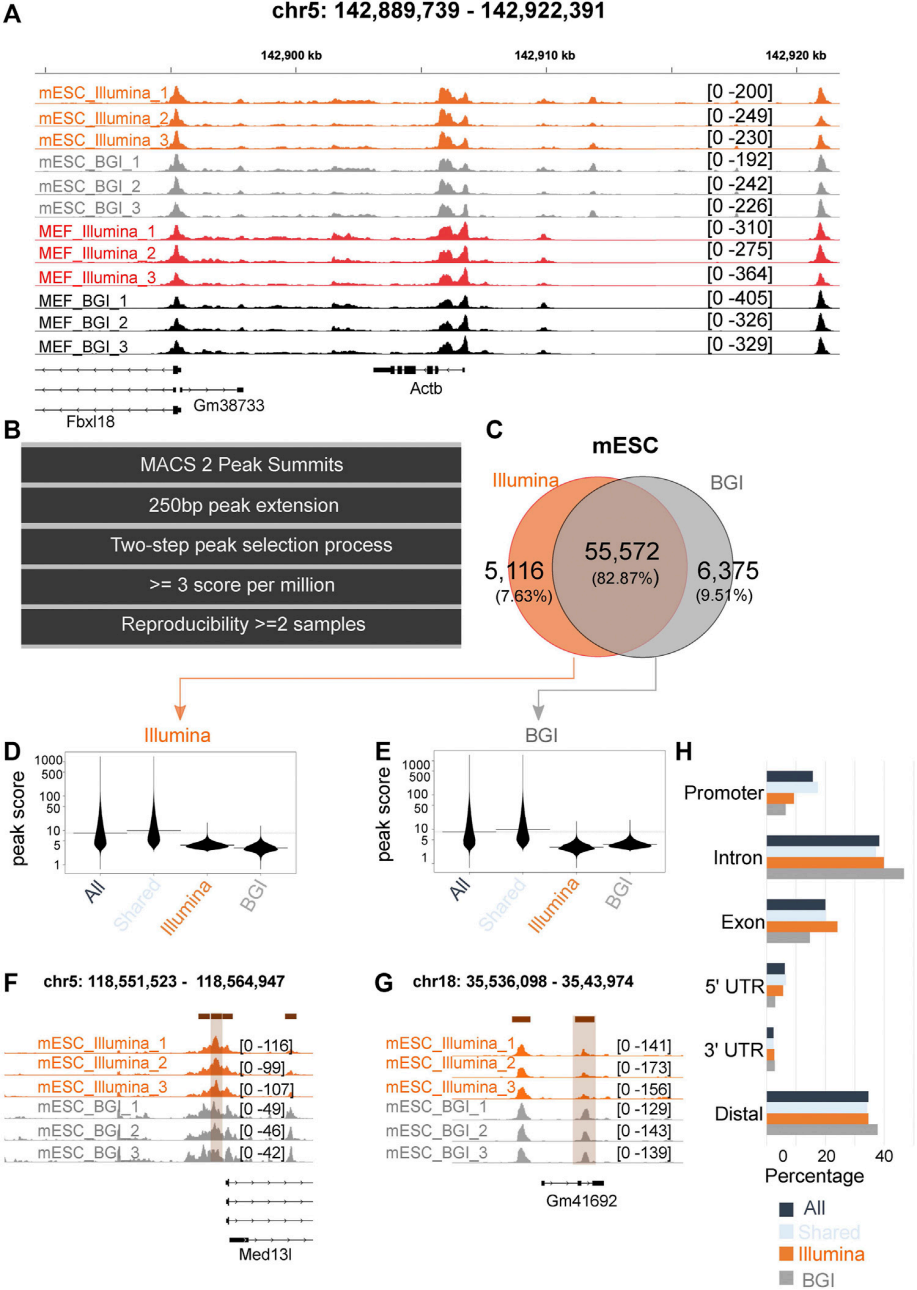
Comparison of peaks identified in data from both platforms. **(A)** Tracks from all samples at the locus of the Actb housekeeping gene. **(B)** Computational workflow to identify high-confidence peaks across the samples of one experimental group (e.g., for three biological MEF replicate samples sequenced on the Illumina platform). **(C)** Venn diagram for high-confidence peaks (across the biological replicates) identified in mESC data sequenced on Illumina and BGI platform. **(D,E)** Peak scores of Illumina and BGI specific peaks relative to the scores of all peaks in the data set and relative to peaks shared by data sets. **(F,G)** Tracks of representative Illumina and BGI specific peaks. **(H)** Genomic context of high-confidence mESC peaks (displayed for all peaks, peaks that are shared/overlap between both data sets, and high-confidence peaks only identified in the Illumina or BGI data set).

By visual inspection, data sets from both platforms produced highly similar peak landscapes as exemplified for the gene locus of *Actb* (Figure 2A). Therefore, we computationally quantified high confidence, reproducible peaks clearly above background levels in each data set. To this end, we only retained peaks where a peak was called in at least two of its three biological replicate samples with a stringent quality cut-off of above three scores per million (a normalized score value that corrects the original peak calling scores for sample sequencing depth and quality). This resulted in a total of 55,071 and 58,813 peaks for MEFs and 60,956 and 62,270 peaks for mESCs for Illumina and BGI, respectively, and a total of 61,981 and 67,063 peaks in MEFS and mESCS identified by at least one sequencing platform (Figures 2B,C; Supplementary Figure 2A).

Out of the high-confidence peaks identified for MEFs, 83.14% overlapped between both platforms (Supplementary Figure 2A); while 82.87% of peaks identified for mESC overlapped between the BGI and Illumina data sets (Figure 2C). The number of peaks only identified by the BGI sequencer resulted in 7,051 and 6,375 peaks for MEFs and mESCs and representing 11.39% and 9.51% of total peaks. On the other hand, peaks only identified by the Illumina sequencer numbered 3,384 and 5,116 peaks, representing 5.47% and 7.63% of total peaks for MEFs and mESC, respectively.

We investigated the original peak scores of the classified peaks (all peaks, shared peaks, and BGI and Illumina-specific peaks as per Figures 2B, C and Supplementary Figures 2B,C). Peaks found in data from both platforms had on average a higher-peak score compared to BGI and Illumina-specific peaks (Figures 2D–G; Supplementary Figures 2B–E). Conversely, BGI and Illumina-specific peaks had lower average peak scores. Importantly they tended to be called in data from both sequencing platforms, but due to lower peak scores, they did not make our stringent cut-offs for high confidence and reproducibility across biological replicates for one of the platform’s data (Figures 2D–G; Supplementary Figures 2B–E). BGI platform-specific peaks (unlike Illumina-specific peaks) tended to occur at increased frequency in distal elements (Figure 2H; Supplementary Figure 2F) at the expense of exonic elements in relation to the element mapping of all the peaks collectively detected in both data sets (Figure 2H; Supplementary Figure 2F).

Cumulatively, we were able to recover 1,314 more high-confidence peaks for MEFs and 3,742 more peaks for mESCs in the case of the BGI data set. This is likely related to differences in insert size distribution, with a higher percentage of small fragments in BGI data (Figures 1C–E; Supplementary Table 2), which we believe leads to slightly sharper peak bases. In support of this, BGI-specific high-confidence peaks on average had both increased peak height and narrower peak bases relative to Illumina data (Supplementary Figure 3). Conversely, Illumina-specific peaks only had higher peak summits, while displaying broader peak bases compared to the matching BGI data peaks (Supplementary Figure 3). To provide direct evidence that differences in insert size affect peak calling quality, we individually mapped sub-nucleosomal reads or a matched number of bi-nucleosomal reads (mESC samples) to the reference genome followed by peak calling. While the number of peaks called for pure sub-nucleosomal reads or pure bi-nucleosomal reads was comparable, on average peaks derived from sub-nucleosomal reads had more significant *p*-values (Supplementary Figure 4A) and a considerably sharper peak shape (Supplementary Figure 4B). This supports the notion that the use of data with a higher proportion of sub-nucleosomal reads can be advantageous in a peak calling context.

### Comparable Motif Enrichment and TF Foot-Printing Performance for Data From Both Platforms

**FIGURE 3:**
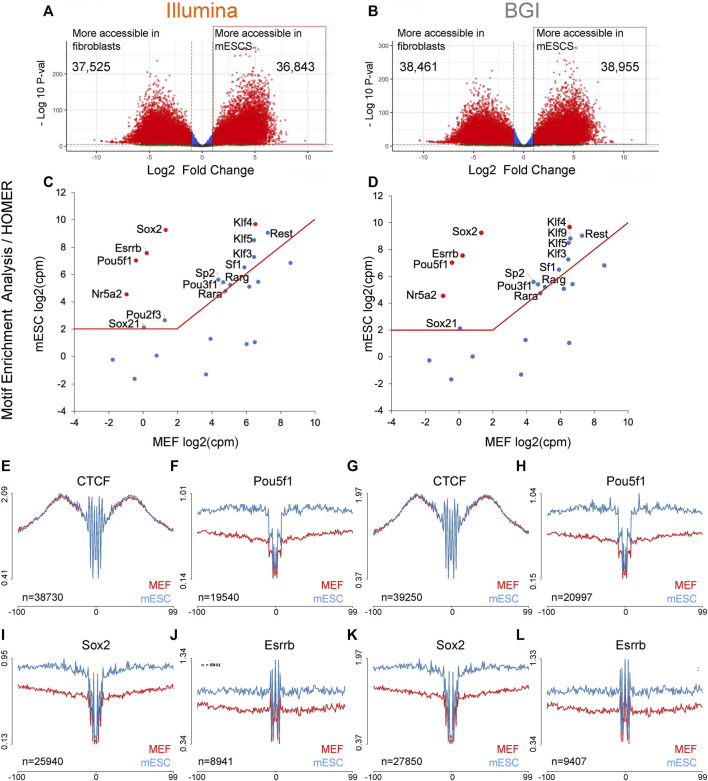
Motif enrichment analysis to recover TF that underpins the mESC identity. **(A,B)** Volcano plots for differentially accessible peaks between MEFs and mESCs were identified for data from both platforms (*n* = 3 biological replicates). **(C,D)** The top 25 motifs identified by HOMER with higher enrichment in mESC specific regions were integrated with transcriptional data for mESCs and MEFs (*n* = 3 biological replicates) to visualize expression differences of linked TFs across both cell types. The top five motifs (re-ranked by expression differences) are indicated in red. Both data sets recovered the same five TF (indicated in red) containing three published 3-factor combinations previously demonstrated to enable pluripotency induction (Pou5f1, Sox2, Klf4; Pou5f1, Esrrb, Klf4, Nr5a2, Sox2, Klf4). **(E–L)** Aggregate TF footprint profiles for CTCF, Pou5f1, Sox2, and Esrrb for both Illumina and BGI ATACseq data sets (profiles are based on the merged data of three biological replicates). The number of TFBS used to generate each aggregate is indicated in the bottom left corner of each plot and is based on PMW scanned sites that intersect with a sample’s open chromatin regions.

In addition to the identification of regulatory elements through open chromatin regions, another key use of ATAC-seq data is the identification of TFs that underpin the differences between cell states of interest (Neto et al., 2017; Dobreva et al., 2018; Alexandre et al., 2021). Methods for this include motif enrichment analysis [e.g., HOMER (Heinz et al., 2010)], operating on peaks that are more accessible in the cell states of interest to infer over-represented TF motifs. Computational pipelines have also been devised recently to assess whether TF binding sites within open chromatin regions are physically occupied in a site-specific manner [e.g., HINT-ATAC (Li et al., 2019), TOBIAS (Bentsen et al., 2020)]. As the fibroblast to pluripotent stem cell transition is well characterized, with most if not all key TFs that underpin the pluripotent state known, this is an ideal system for benchmarking analyses. To identify motifs of TF with increased activity in mESCs relative to fibroblasts, we determined peaks with higher accessibility in mESCs relative to MEFS and used them for motif enrichment analysis (HOMER, known motifs) using peaks with higher accessibility in MEFs as background peak set (Figures 3A,B).

The top 25 non-redundant TF motifs (ranked by false discovery rate [FDR]; Supplementary Table 3) were integrated with a transcriptional data set already present in the laboratory for mESCs and MEFs (note: transcriptional data was sequenced on an Illumina platform as our study’s focus is the comparison of ATAC-seq data from different platforms). Key TFs that underpin the mESC identity are expected to be both robustly expressed in mESCs (>2 log2-transformed counts per million [log2CPM]) and at higher levels than in MEFs. To visualize the expression levels of each TF within the top 25 non-redundant motifs, their log2CPM values in both cell types were graphed against each other (Figures 3C,D). For both Illumina or BGI derived data, the top five TFs (re-ranked by expression differences between MEFs and mESCs) were Pou5f1 (=Oct4), Sox2, Esrrb, Nr5a2, and Klf4, containing three published 3-factor combinations (Pou5f1/Sox2/Klf4; Pou5f1/Esrrb/Klf4; Nr5a2/Sox2/Klf4) that have been shown to enable pluripotency induction (Nakagawa et al., 2008; Feng et al., 2009; Heng et al., 2010). This indicates a comparable ability for data from both platforms to directly recover known reprogramming factors based on motif enrichment analysis.

In addition to predicting TF activity changes via motif enrichment analysis, ATAC-seq data, especially in the case of OMNI-ATAC-seq data, due to its low background levels, can also be used to assess whether TFBS within open chromatin are occupied by generating aggregate foot-print profiles across all the binding sites of specific TFs of interest. Since TF occupied sites are protected from Tn5 adapter insertion, occupied sites display a local drop in Tn5 insertion events, which can be visualized via aggregate profiles across all scanned TFBS within open chromatin regions (e.g., after correction for Tn5 cleavage bias using a position dependency model as per the HINT-ATAC pipeline (Li et al., 2019)). We assessed data from both instruments for their ability to visualize aggregate accessibility profiles across TFBS determined via position weight matrix (PWM) scanning in the absence of filtering for occupied sites via site-specific foot-printing (note: the HINT-ATAC pipeline was used to correct for Tn5 cleavage bias for these initial analyses). Aggregate profiles for ubiquitous TF CTCF binding sites showed comparable profiles across both cell types and platforms, while aggregate footprints for Pou5f1, Sox2, and Esrrb showed, as expected, higher occupancy status in mESCs relative to MEFs (Figures 3E–L). The only observable difference between data from both platforms was that marginally more TFBS intersected with accessible chromatin and were, therefore, used for visualization for BGI data as more peaks could be called for data from this platform (Figures 3E–L, e.g., 19540 vs. 20997 TFBS for Pou5f1 for Illumina and BGI data, respectively).

Pipelines like HINT-ATAC (Li et al., 2019) or TOBIAS (Bentsen et al., 2020) can also be used to infer whether a TFBS, at a specific genomic locus, is occupied or not based on the absence or presence of local Tn5 insertion events. HINT-ATAC determines global differences in TF activity between two cell states by restricting analysis of aggregate footprinting to TFBS with evidence for physical occupancy in one of the cell states (Li et al., 2019). HINT-ATAC then quantifies differences in TF activity based on differences in depth of aggregate footprints. Conversely, the TOBIAS pipeline merely determines the probability that specific sites are occupied or not and then compares differential binding between biological conditions (Bentsen et al., 2020). Using both pipelines, we quantified TF activity/binding differences between mESCs and MEFs. This was followed by transcriptional integration (as per Figures 3C,D) of the top 40 non-redundant TF candidates with higher activity in mESCs (Supplementary Tables 4, 5).

**FIGURE 4:**
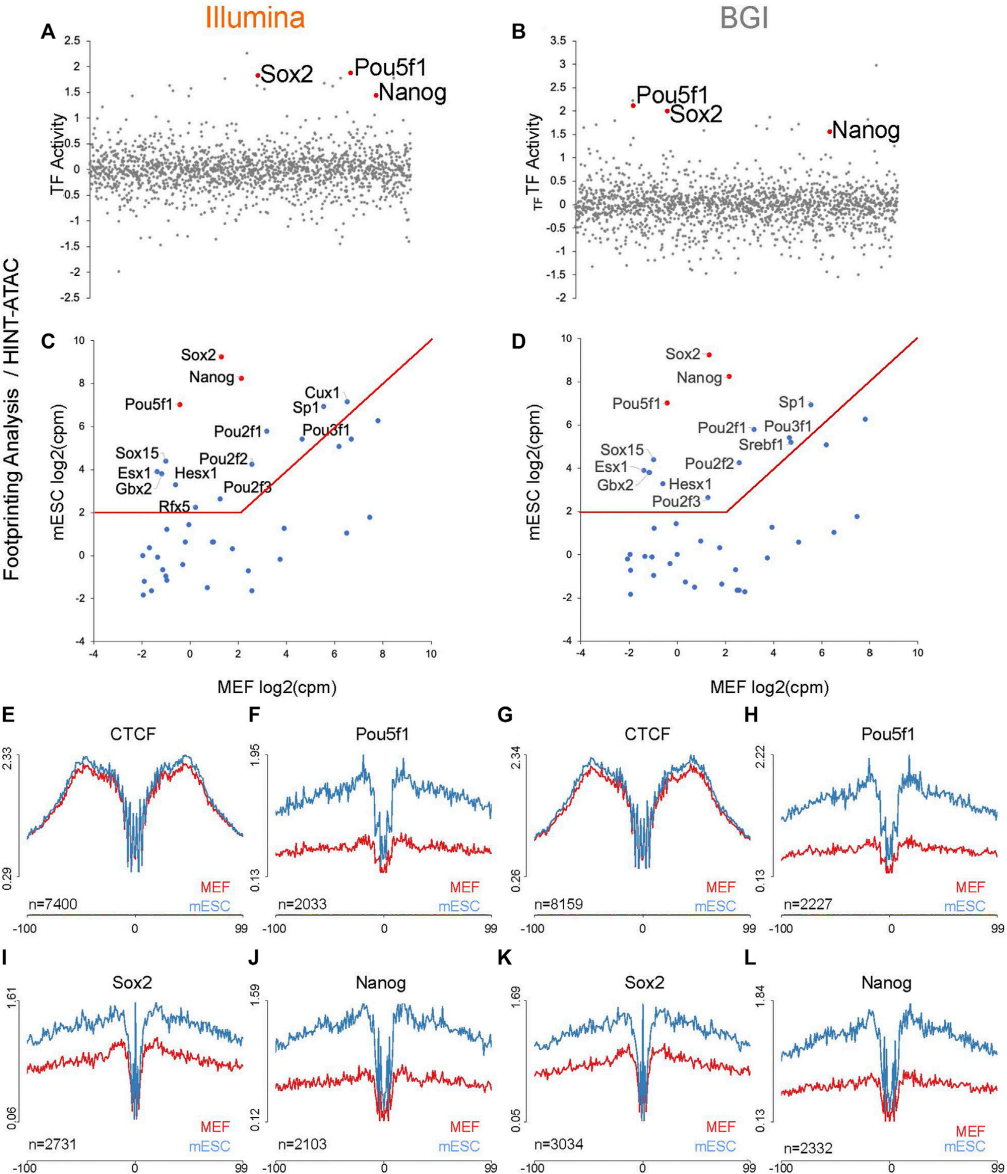
Foot-printing analysis with Hint-ATAC pipeline to recover TF that underpins the mESC identity. **(A,B)** TF activity changes quantified by Hint-ATAC pipeline for mESC vs. MEFs, position of Pou5f1, Nanog, and Sox2 are indicated in red (analysis is based on the merged data of three biological replicates as HINT-ATAC does not accept replicate data). **(C,D)** The top 40 motifs identified by Hint-ATAC with higher activity in mESC were integrated with transcriptional data for mESCs and MEFs (*n* = 3 biological replicates) to visualize expression differences of linked TFs across both cell types. The top three motifs (re-ranked by expression differences) are indicated in red. **(E–L)** Representative aggregate accessibility profiles of TFBS with evidence for occupancy in the cell types for CTCF, Pou5f1, Sox2, and Nanog (profiles are based on the merged data of three biological replicates). The number of TFBS used to generate each aggregate is indicated in the bottom left corner of each plot and is based on PMW scanned sites (with evidence for occupancy in one of the cell types) that intersect with a sample’s open chromatin regions.

The top three TFs recovered by the HINT-ATAC pipeline (re-ranked by expression differences between MEFs and mESCs) are known reprogramming factors capable of inducing pluripotency together (Pou5f1, Sox2, and Nanog (Yu et al., 2007), and these factors were retrieved for data from both platforms (Figures 4A–D). As expected, aggregate profiles for TFBS, filtered for binding sites with evidence for physical occupancy by HINT-ATAC had lower background levels for data from both platforms (i.e., Pou5f1 and Nanog TFs are not expressed in MEFs and as such their TFBS should not be occupied) (Figures 4E–L).

**FIGURE 5:**
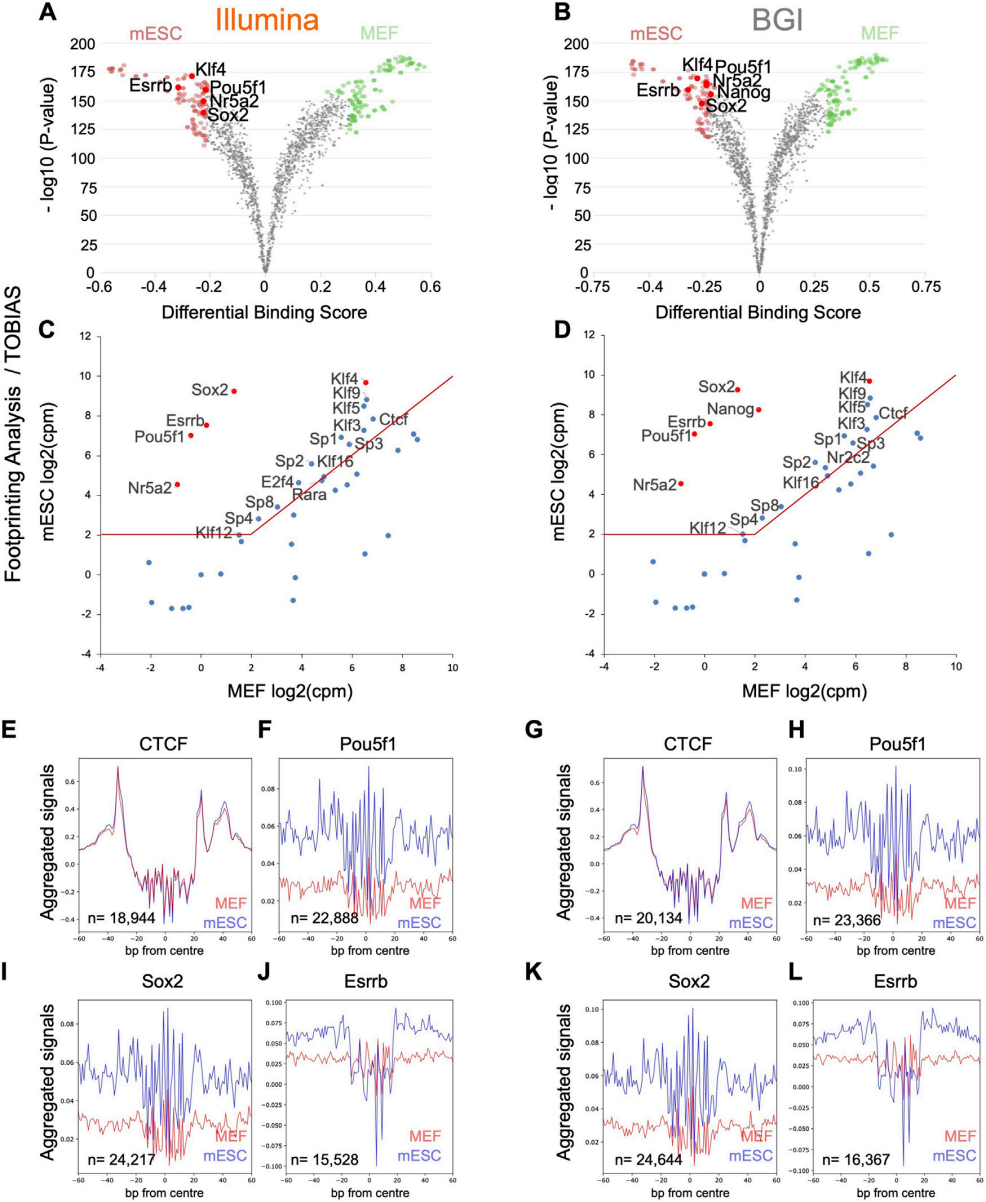
Foot-printing analysis with TOBIAS pipeline to recover TF that underpins the mESC identity. **(A,B)** TF binding changes quantified by TOBIAS pipeline are visualized in the form of volcano plots (analysis is based on the merged data of three biological replicates as TOBIAS does not accept replicate data). **(C,D)** The top 40 motifs identified by TOBIAS with higher binding activity in mESC were integrated with transcriptional data for mESCs and MEFs (*n* = 3 biological replicates) to visualize expression differences of these TFs across both cell types. The top five to six motifs (re-ranked by expression differences) are indicated in red containing three published 3-factor combinations previously demonstrated to enable pluripotency induction (Pou5f1, Sox2, Klf4; Pou5f1, Esrrb, Klf4; Nr5a2, Sox2, and Klf4). **(E–L)** Representative aggregate accessibility profiles of TFBS with evidence for occupancy in one of the cell types for CTCF, Pou5f1, Sox2, and Nanog (profiles are based on the merged data of three biological replicates). The number of TFBS used to generate each aggregate is indicated in the bottom left corner of each plot that intersect with a sample’s open chromatin regions.

In contrast to HINT-ATAC, transcriptional integration of the top 40 TFs with higher binding activity in mESC determined by TOBIAS (Supplementary Table 5; Figures 5A,B) retrieved among the top six factors (re-ranked by expression differences between MEFs and mESCs) Pou5f1, Sox2, Esrrb, Nr5a2, and Klf4. These factors were also recovered by HOMER (Figures 3C,D) and contained three published 3-factor combinations each shown to induce pluripotency (Heng et al., 2010; Feng et al., 2009). Pou5f1, Sox2, Esrrb, Nr5a2, and Klf4 were equally detected through data from both platforms. In addition, BGI data recovered Nanog, another known reprogramming factor (Yu et al., 2007) among its top six candidates, indicative of a slight increase in sensitivity when using this data type.

While the TOBIAS pipeline was better at recovering verified reprogramming factors through its top 40 candidates (Figures 5C,D) compared to HINT-ATAC (Figures 4C,D), interestingly HINT-ATAC produced sharper aggregate foot-printing profiles (Figures 4E–L) compared to TOBIAS (Figures 5E–L).

Finally, we assessed how data generated by both sequencing platforms performed at recovering ChiP-seq-verified TFBS from published ENCODE data. Our assessment showed comparable enrichment of TFBS for CTCF, Pou5f1, and Nanog (ChiP-seq-verified in mESC) and CTCF (ChiP-seq-verified in MEFs) in open chromatin, with a consistent trend for improved performance in BGI derived data (Supplementary Figure 5).

Collectively, our analyses indicate that data from both platforms can be used for TF foot-printing-based analyses at overall comparable levels.

## Discussion

ATAC-seq-based chromatin state accessibility studies are widely used for epigenetic characterization on a bulk and single-cell level (Minnoye et al., 2021). Despite the importance of this assay, no investigation has been conducted to compare the performance of the highly economical BGI sequencing technology relative to the generally employed Illumina technology for this critical library type. Here we complement published knowledge about BGI sequencing technology by showing that it cannot only generate data for RNA-seq (Senabouth et al., 2020; Natarajan et al., 2019) and whole-genome DNA libraries (Mak et al., 2017; Zhu et al., 2021) but also for ATAC-seq libraries at levels comparable to an Illumina platform. More specifically BGI’s DNBSEQ-G400 instrument enabled the generation of chromatin accessibility data that could be used to identify master TFs that underpin cell identity, with motif enrichment, TF aggregate and *de novo* foot-printing capabilities equivalent to data from an Illumina platform. The only noteworthy difference was that the same libraries sequenced on either platform displayed differences in insert fragment size distribution. This is in accordance with a previous study comparing both technologies for whole-genome sequencing performance (Mak et al., 2017), which also observed that BGI instruments were biased toward recovering reads with shorter insert sizes. We conjecture that either the nanoball library conversion step or preferential clustering of nanoballs with shorter inserts on DNBSEQ-G400 lanes results in a higher proportion of shorter fragments being sequenced relative to the Illumina instrument. Enrichment of DNBSEQ-G400-derived data for sub-nucleosomal reads resulted in slightly narrower peak bases, which enabled marginally better peak calling and the identification of >4% more differentially accessible peaks between MEFs and mESCs. While this increase in peak calling sensitivity had no noticeable effect on data interpretation, it is possible that the DNBSEQ-G400 platform might significantly outperform the Illumina instrument in the case of under-tagmented ATAC-seq samples where recovery of more sub-nucleosomal reads at the expense of larger fragments could have a marked effect on data quality. It is also conceivable that new techniques, such as CUT&Tag (Kaya-Okur et al., 2019), increasingly performed instead of ChiP-Seq, and also based on Tn5-mediated DNA fragment insertion, might benefit from sequencing on the DNBSEQ-G400, in particular when targeting TFs where the recovery of short fragments is of particular interest.

While not the main focus of our study, it is noteworthy that integrative analysis of OMNI-ATACseq data (generated via both platforms) with transcriptional data performed extremely well at identifying master TFs that underpin the pluripotent cell identity relative to the somatic state. The top non-redundant TF candidates (re-ranked by expression differences between mESCs and MEFS) put forward through both HOMER and the TOBIAS pipeline contained each at least three published three-factor combinations that can induce pluripotency (Heng et al., 2010; Feng et al., 2009). Hence, the use of low-noise OMNI-ATAC-seq data for quantification of global changes in TF activity, in conjunction with basic RNA-seq data integration offers a surprisingly effective and direct way to identify master regulators known to drive the somatic to pluripotent cell state transition compared to approaches using network-based analyses (Jung et al., 2021; Kamaraj et al., 2016). Thus, we confirm that chromatin state data is an excellent basis to recover reprogramming factors (Hammelman et al., 2022) and in addition demonstrate in this study that effective recovery hinges on integration with RNAseq data. Another notable observation was that aggregate foot-printing based on HINT-ATAC produced clearer profiles relative to the TOBIAS pipeline, indicating that HINT-ATAC’s PDM might be better at correcting for the Tn5 insertion bias. Since TOBIAS is basing its Tn5 insert bias correction on background sequences, not part of the input peak set, its performance might be improved by stringently depleting small peaks from the background, which was not done in this current study. Relative to HINT-ATAC, TOBIAS was better at recovering known TFs that underpin the pluripotent state, implying that its downstream quantification procedure for differential TF binding might be more accurate. Future methods based on correction of the Tn5 cleavage bias through a PDM (like HINT-ATAC’s) followed by quantification of global TF binding changes (like TOBIAS’ approach) might outperform current pipelines.

Overall, our study concludes that DNBSEQ-G400 and HiSeqX10-derived data enabled comparable levels of open chromatin identification for the high-quality OMNI-ATACseq libraries used in this current study, yielding similar analytical outcomes, albeit at low sequencing costs in the case of the BGI instrument.

## Materials and Methods

### OMNI-ATACseq Library Preparation and Sequencing

MEFs and mESCs (all C57/Bl6 background) were cultured as previously described (Firas et al., 2014; Chen et al., 2018). Experimental procedures were approved by the University of Queensland Animal Ethics Committee and carried out in accordance. Antibody labeling workflow and FACS isolation of Thy1+ MEFs and Thy1-/Ssea1+/Epcam+ mESCs were also detailed previously (Nefzger et al., 2015; Chy et al., 2017). OMNI-ATACseq libraries were generated from FACS purified cells according to the standard protocol (Corces et al., 2017). Afterward, libraries were purified using a QIAGEN MinElute PCR purification kit (QIAGEN, Cat#28004) followed by Agencourt AMPure XP beads (Beckman Coulter, Cat#A63880) according to the manufacturer’s recommendations. Library fragments ranging from 150 to 700 bp were enriched and the final elution volume was 21 ul. The finalized libraries were provided to BGI for sequencing. HiSeqX10 sequencing was performed in 150 bp PE mode followed by read trimming to 100 bp PE for analysis. In case of sequencing on the DNBSEQ-G400 instrument, libraries were converted to nanoballs, as part of BGI’s sequencings service, and sequenced according to established workflows in 100 bp PE mode (Senabouth et al., 2020).

### ATAC-Seq Data Processing and Alignment

All sequencing data were analyzed using the GRCm38.p6/mm10 mouse reference genome. All genomic comparisons were performed on the whole genome excluding chromosome Y.

ATAC-seq data processing and alignment were completed using the Harvard pipeline (https://informatics.fas.harvard.edu/atac-seq-guidelines.html). The GRCm38.p6/mm10 genome primary assembly build used for alignment was obtained from the ensemble database (http://asia/ensembl.org/Musc_musculus/Info/Index; ftp://ftp.ensembl.org/pub/release-101/fasta/mus_musculus/dna/Mus_musculus.GRCm38.dna.primary_assembly.fa). All Illumina 150 bp fastq files were trimmed to 100 bp. Next, all fastq files were trimmed to remove the Illumina Nextera Transposase adapter sequence using Cutadapt v.2.4 with “-m 20” parameter (Martin, 2011). After trimming, FastQC v0.11.8 (https://www.bioinformatics.babraham.ac.uk/projects/fastqc/) was used to check overall sequence quality and evaluate proper adapter trimming. Bowtie2 (Langmead and Salzberg, 2012) was used to align reads to the GRCm38.p6/mm10 mouse reference genome using “-p 8, –very-sensitive” options. Picard tools (http://broadinstitute.githyb.io/picard/) were used to mark and remove duplicates using the MarkDuplicates tool with default options. All subsequent analyses were performed on deduplicated reads. Samtools (Li et al., 2009) was used to sort and obtain uniquely mapped reads using “-b -q 10” options. Samtools were also used to remove reads from the mitochondrial chromosome. Aligned, deduplicated BAM files were down-sampled using picard tools (http://broadinstitute.githyb.io/picard/) *DownSamp* with the corresponding P parameter to obtain 60 million reads for MEF samples and 45 million reads for mESC samples. Down-sampled files were used for downstream analysis. For visualization purposes, BAM files were converted to an index binary format bigWig using bedtools (Quinlan, 2014).

### Peak Calling

Peak calling was performed to ensure high-quality fixed-width peaks in accordance with Corces et al. (2018). For each sample, peak calling was performed using the MACS2 callpeak command with the following parameters “-g mm –shift -100 –extsize 200 –nomodel –call-summits –nolambda –keep-dup all -p 0.01” (Zhang et al., 2008; Feng et al., 2012). Then, peak summits were extended by 250 bp on both sides to a final width of 501 bp. Peaks were filtered for mm10 blacklisted regions (Amemiya et al., 2019) (https://www.encodeproject.org/annotations/ENCSR636HFF/).

Consensus matrices were created similarly to the approach described in Corces et al. (2018) consisting of a two-step process. Firstly, overlapping peaks within the same sample (as a result of the 250 bp peak summit extension) were handled using an iterative removal approach. In other words, in the case of overlapping peaks within the same sample, only the most significant peak was kept. This process was performed in an iterative manner so that each peak within a sample was considered individually to be kept or removed based on their overlap and significance score. This process resulted in a set of fixed-width peaks per sample.

The remaining significance peak scores “[−log10 (*p*-value)]” per sample were converted to score per million values by dividing each individual peak score by the sum of all of the peak scores in the given sample divided by 1 million. This normalized score per million value corrects the original peak calling scores for sample sequencing depth and quality, as higher quality samples yield a higher number of peaks and higher significance scores overall. Thus, scores per million allow the direct comparison of peaks across biological replicates required for the second step of the process.

Secondly, to generate consensus matrices across conditions MEFs, mESCs, and sequencing platform (BGI or Illumina), we generated a cumulative peak set per cell type and sequencing platform, and then for peaks overlapping across samples only the one with the highest score per million was maintained. Only peaks with a score per million value ≥3 observed in at least two samples (minimal overlap 50%) were further considered. This resulted in a set of fixed-with, reproducible, high-quality peaks per cell type and sequencing platform for MEFs of 58,813, and 55,071 for BGI and Illumina sequencing platforms and for mESCs of 62,270 and 60, 956 for BGI and Illumina sequencing platforms, respectively.

In addition, we also generated a cell-type-specific consensus peak set representing reproducible peaks from MEFs and mESCs found in either sequencing platform (BGI and Illumina). For this, we re-normalized the score per million scores for each cell type and platform-specific consensus peak set to avoid over-representation of peaks from cell types with higher depth or quality. Then, the iterative peak overlapping and removal approach was performed resulting in a final peak set of 61,891 for MEFs and 67,063 for mESCs. These peak sets were used to determine the percentage of peak overlap between sequencing platforms per mESCS (Figure 2) and MEFS (Supplementary Figure 2).

Lastly, we also generated a sequencing platform peak set representing reproducible peaks from both cell types (mESCs and MEFs). This final consensus peak set contained 91,355 and 87,028 for the BGI and Illumina sequencing platforms, respectively. Peaks per sequencing platform were used to obtain differential accessible regions between cell types per sequencing platform followed by motif discovery (Figure 3).

### ATAC-Seq Data QC -Transcription Start Site Enrichment, Fragment Length Distribution, FROT, FRIP

The fraction of reads overlapping TSS (FROT) is a measure of signal-to-noise ratio and therefore data quality in ATAC-seq data sets (Gorkin et al., 2020). We calculated FROT for each library as the number of reads that map within 1 KB of protein-coding gene annotation of the mouse genome (GRCm38), version M25 (Ensembl 100), divided by the total number of usable reads. In addition, to visualize the TSS enrichment per profile, we made use of deeptools v 3.3.1 computeMatrix reference-point. Next, the TSS enrichment profiles were subsequently plotted using deeptools plotHeatmap (Ramírez et al., 2014).

The fragment length distribution was plotted using Picard tools ColectInsertSizeMetics (http://broadinstitute.github.io/picard/), where the insert size is the distance between the R1 and R2 read pairs, indicating the size of the DNA fragment the read pairs came from.

The fraction of reads in peaks (FRIP) was calculated as the number of reads mapping in called peaks by MACS2 narrow peak and peaks with a score per million ≥3 per sample. Peaks were considered “distal” if they did not overlap ±1,000 bp from annotated TSSs. Proximal peaks were considered as that overlapping ±1,000 bp from annotated TSSs. Reads not overlapping distal or proximal peaks were classified as “not in peaks.”

### Overlap With Genomic Features

Genomic features, exonic, intronic, 5′ UTR, 3′UTR, promoter, and distal, were derived from the mouse genome (GRCm38), version M25 (Ensembl 100). Promoter regions were considered as those within ±1,000 bp from annotated TSSs. Overlap of reads with genomic features was determined using bedtools intersect and considered to overlap if 40% of the reads fell within a genomic feature category (Quinlan, 2014).

### Differential Peak Score Analysis

For the cell-type-specific peak sets combining peaks from both sequencing platforms with 61,891 for MEFs and 67,063 for mESCs peaks, we gathered their original peak score given per sequencing platform through the intersection with the platform-specific peaks for Figures 2C–H and Supplementary Figure 2. This analysis was performed with reproducible peaks with a score per million ≥3 score per million in each sequencing platform and the original dataset containing ≥0 score per million. The score per million values for different peak categories, all, shared and platform specific, BGI and Illumina were visualized. This resulted in 1,227 and 753 peaks for Illumina and BGI data, respectively not being called due to reproducibility stringency.

### Differential Accessibility Analysis

The number of reads falling within accessible regions was calculated using the package subread (Liao et al., 2019). Next, differentially accessibility between conditions was evaluated using the R package DESeq2 (Love et al., 2014).

### Motif Enrichment Within Differential Accessible Regions

HOMER v 4.11 (Heinz et al., 2010) (http://homer.ucsd.edu/homer/) motif discovery algorithm (findMotfisGenome.pl) was used to determine motif enrichment (known motifs) in differential accessible regions between cell types. The analyses were performed using input regions with significantly higher accessibility in one cell type (*p*-value < 0.05) and as a background set “--bg” the regions with significantly higher accessibility in the second cell type (*p*-value < 0.05) were used.

### Foot-Printing Analysis Using HINT-ATAC

To identify bound transcription factor binding sites per cell type and sequencing platform, we used the HINT-ATAC (Hmm-based identification of transcription factor footprints) pipeline as described (Li et al., 2019) within the set of high-quality peaks per cell type and sequencing platform, corresponding to 58,813, and 55,071 regions for MEFs per BGI and Illumina sequencing platforms and 62,270 and 60, 956 for mESCS per BGI and Illumina sequencing platforms, respectively. Since HINT-ATAC does not allow the use of replicates, data for the three biological MEF and the three biological mESC replicates were merged for analysis. The pipeline performs a Tn5 bias correction of the ATAC alignment file using their position dependency model (PDM) with a k = 8. The program rgt-hint footprint with the following parameters “--atac-seq and --paired-end --organism = mm10” was performed to identify the genomic locations of the footprints per sample. Next, we matched motifs falling within predicted footprints using the command “rgt-motifanalysis matching”--organism = mm10′ using an extensive collection of 2177 PWMS (Vierstra et al., 2020) gathered from the HOCOMOCO v11 CORE collection for human and mouse, Jaspar 2018 Vertebrate CORE collection and Taipale 2103 HT-SELEX (Jolma et al., 2013). This collection was used in accordance with the ENCODE3 TF foot-printing analysis in human tissues and cell types. Finally, to assess differential activity between cell types, we performed “rgt-hint differential”, resulting in a list of the most differentially active TFs and aggregate foot-printing plots per TF motif. Aggregate plots from ATAC-seq accessible regions in the absence of foot-printing were also generated using “rgt-motifanalysis matching” and “rgt-hint differential” commands for the panels presented in Figures 3E–L.

### Foot-Printing Analysis Using TOBIAS

In addition to HINT-ATAC, we used another method to predict transcription factor binding at footprint resolution, TOBIAS (Transcription factor Occupancy prediction By Investigation of ATAC-seq signal) (Bentsen et al., 2020). Like HINT-ATAC, TOBIAS does not allow the use of replicate data, hence we merged the three biological MEF and the three biological mESC replicates for this analysis. Next, the same set of high-quality peaks per cell type and sequencing platform was used for analysis, corresponding to 58,813, and 55,071 regions for MEFs per BGI and Illumina sequencing platforms and 62,270 and 60, 956 for mESCS per BGI and Illumina sequencing platforms, respectively. The TOBIAS framework initially corrects Tn5 cut signal bias through the tool ATACorrect. In brief, it generates a dinucleotide weight matrix representing the preference of Tn5 insertion using mapped reads from closed chromatin and a background model by shifting reads +100 bp. Finally, reads within open chromatin peaks are corrected by estimating the number of cuts per base pair and subtracting that number from the observed cuts. Here peaks per platform across cell types were merged and the foot-printing correction was performed in both cell type signals in accordance with the TOBIAS procedure. Next, we performed ScoreBigwig which considers regions’ accessibility and corrected the Tn5 signal to provide a foot-printing score per region. Finally, the differential binding between conditions was assessed using the tool BINDETECT where we used the same collection of 2,177 PWMS that was used in conjunction with the HINT-ATAC pipeline.

### Transcriptional Data

RNA-seq libraries were generated and sequenced as previously described (Sun et al., 2021). Raw reads were demultiplexed and trimmed and checked for quality using Sabre (https://github.com/serine/sabre), Cutadapt (Martin, 2011), and FASTQC software (https://www.bioinformatics.babraham.ac.uk/projects/fastqc/). Processed data were aligned to the mouse reference genome (mm10) using the STAR aligner (version 2.5.2b) (Dobin et al., 2013) with the following parameters *--outSAMunmapped Within --outFliterMatchNminOverLread 0.3 --outSAMunmapped Within --outFilterMatchNminOverLread 0.3 --outFilterScoreMinOverLread 0.3 --twopassMode Basic*.

The sorted BAM files were used for UMI collapsing using the function mark dupes within the Je software (version 1.2) (Girardot et al., 2016) with the following parameters *M = 1 READ_NAME_REGEX = null*. The *featurecounts* function within the Subread package (version2.0.1) was used to count uniquely mapped reads to exons (Liao et al., 2019).

## Data Availability

The accession numbers for OMNI-ATAC-seq and whole transcriptome sequencing experiments reported in this article have been made publicly available through Gene expression omnibus. The SuperSeries GSE201578 is composed of the subseries GSE201576 and GSE201577, where RNA-seq and ATAC-seq data are stored, respectively. Requests for further information should be directed to the Lead Contact, CN (c.nefzger@imb.uq.edu.au).

## Supplementary Material

Supplementary Figure S1 Insert size distribution for untruncated HiSeqX10 PE 150 bp data relative to DNBSEQ-G400 100 bp data. **(A,B)** Quantification of percentage of sub-nucleosomal reads in Illumina versus BGI data for **(A)** MEFs and **(B)** mESCs (*n* = 3, biological replicates, Student’s *t*-test, two-tailed, unpaired). **(C,D)** Quantification of the percentage of bi-nucleosomal reads in Illumina versus BGI data for **(C)** MEFs and **(D)** mESCs (*n* = 3, biological replicates, Student’s *t*-test, two-tailed, unpaired).

Supplementary Figure S2 Comparison of peaks identified in MEF data. **(A)** Venn diagram for high confidence peaks (across the biological replicates) identified in MEF data sequenced on Illumina and BGI platform (based on three biological replicates). **(B,C)** Peak scores of Illumina and BGI specific peaks relative to the scores of all peaks in the data set and peaks shared by data sets. **(D,E)** Tracks of representative Illumina and BGI specific peaks. **(F)** Averaged genomic context of high confidence MEF peaks (for all peaks, peaks that are shared/overlap between both data sets, and high peaks only identified in the Illumina or BGI data set).

Supplementary Figure S3 Visualisation of peak sharpness for all, shared, BGI and Illumina specific peaks. Each plot shows the aggregate accessibility profiles of shared, BGI and Illumina specific peaks for each of the three biological replicates.

Supplementary Figure S4 Peak calling with separately mapped subnucleosomal and bi-nucleosomal reads. **(A)** Number of separately mapped sub-nucleosomal and bi-nucleosal mESC reads used for peak calling, number of called peaks and their average *p*-value (based on one biological replicate). **(B)** Visualisation of representative peaks derived from un-fractioned data, separately mapped sub-nucleosomal reads and separately mapped bi-nucleosomal reads. Score per million (a normalized score value that corrects the original peak calling scores for sample sequencing depth and quality) is indicated on the right-hand side of each peak.

Supplementary Figure S5 ENCODE ChiP-seq signal recovery. Visualisation of signal recovery/enrichment of known TFBS for CTCF, Pou5f1 and Nanog (ChiP-seq verified in mESC) and CTCF (ChiP-seq verified in MEFs) in open chromatin for each of the three biological replicates.

Supplementary Table S1 Mapping statistics.

Supplementary Table S2 Insert size distribution.

Supplementary Table S3 Top 25 non-redundant motifs identified via HOMER.

Supplementary Table S4 Top 40 non-redundant motifs identified via Hint-ATAC.

Supplementary Table S5 Top 40 non-redundant motifs identified via TOBIAS.
