# Supplementary figures and images for: Benchmarking of ATAC Sequencing Data From BGI’s Low-Cost DNBSEQ-G400 Instrument for Identification of Open and Occupied Chromatin Regions
Source: Front Mol Biosci. 2022 Jul 7;9:900323. doi: 10.3389/fmolb.2022.900323 (PMC9302965; doi:10.3389/fmolb.2022.900323)

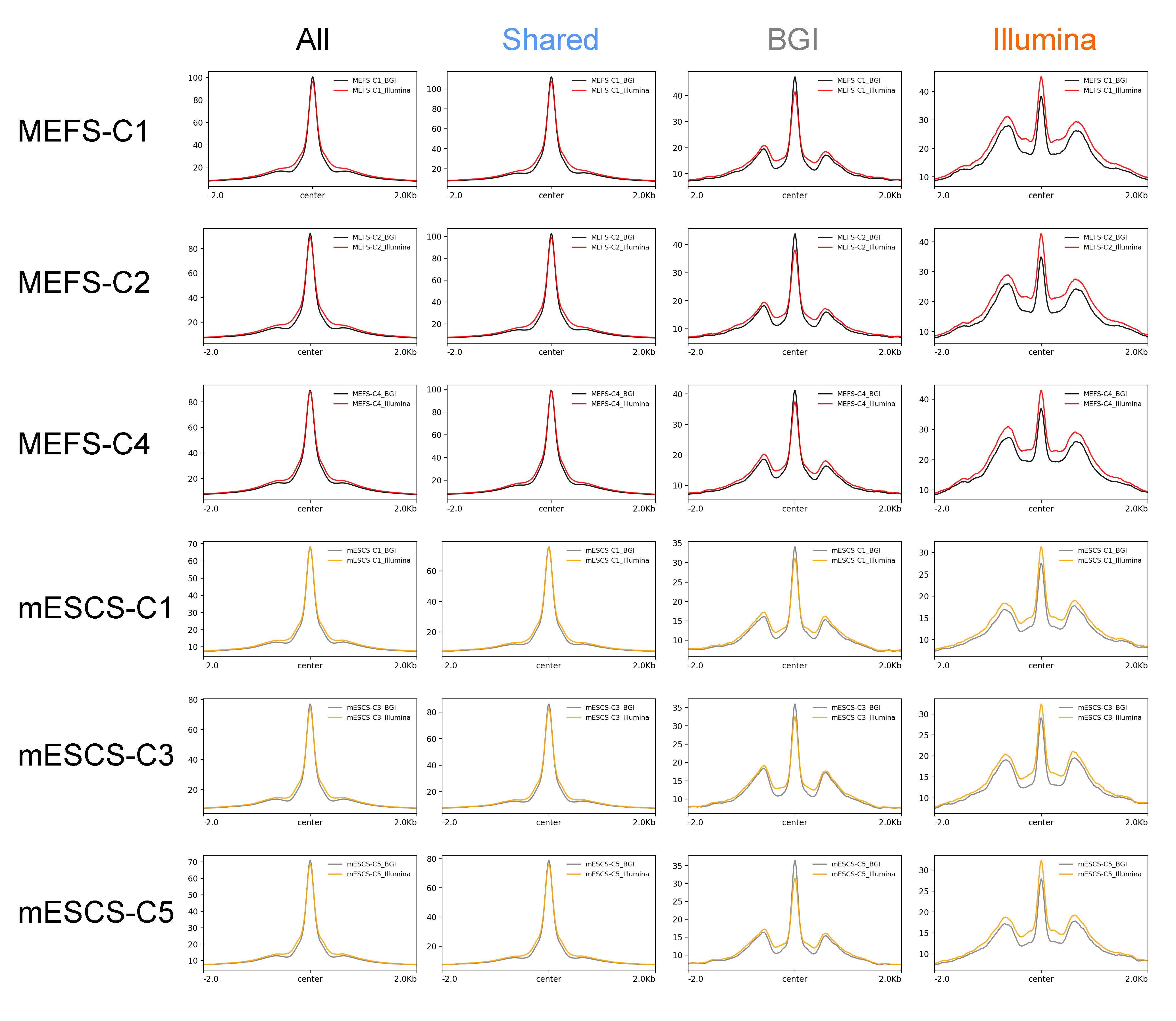

Supplement: Supplementary file 2 [file Image3.JPEG]

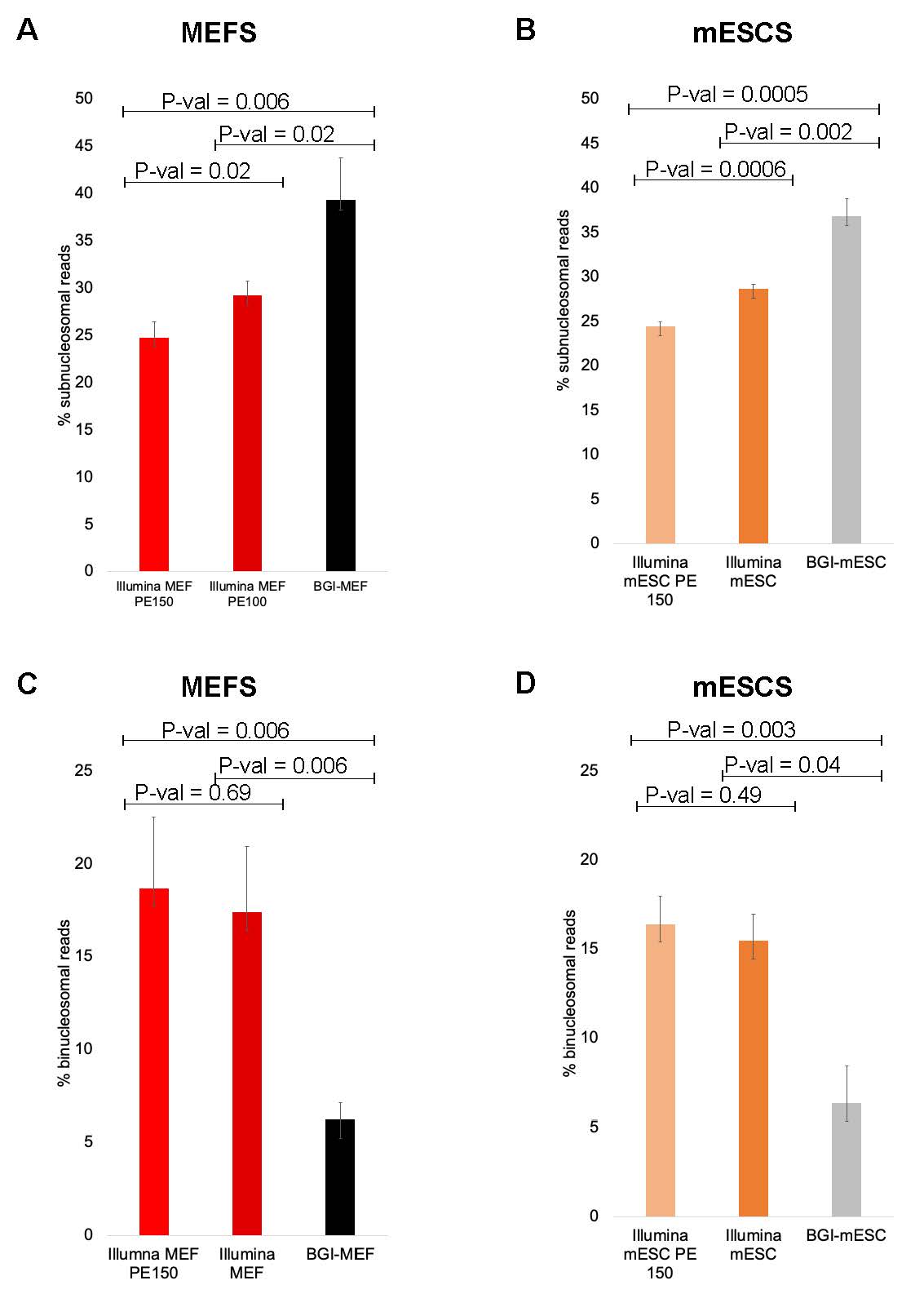

Supplement: Supplementary file 4 [file Image1.JPEG]

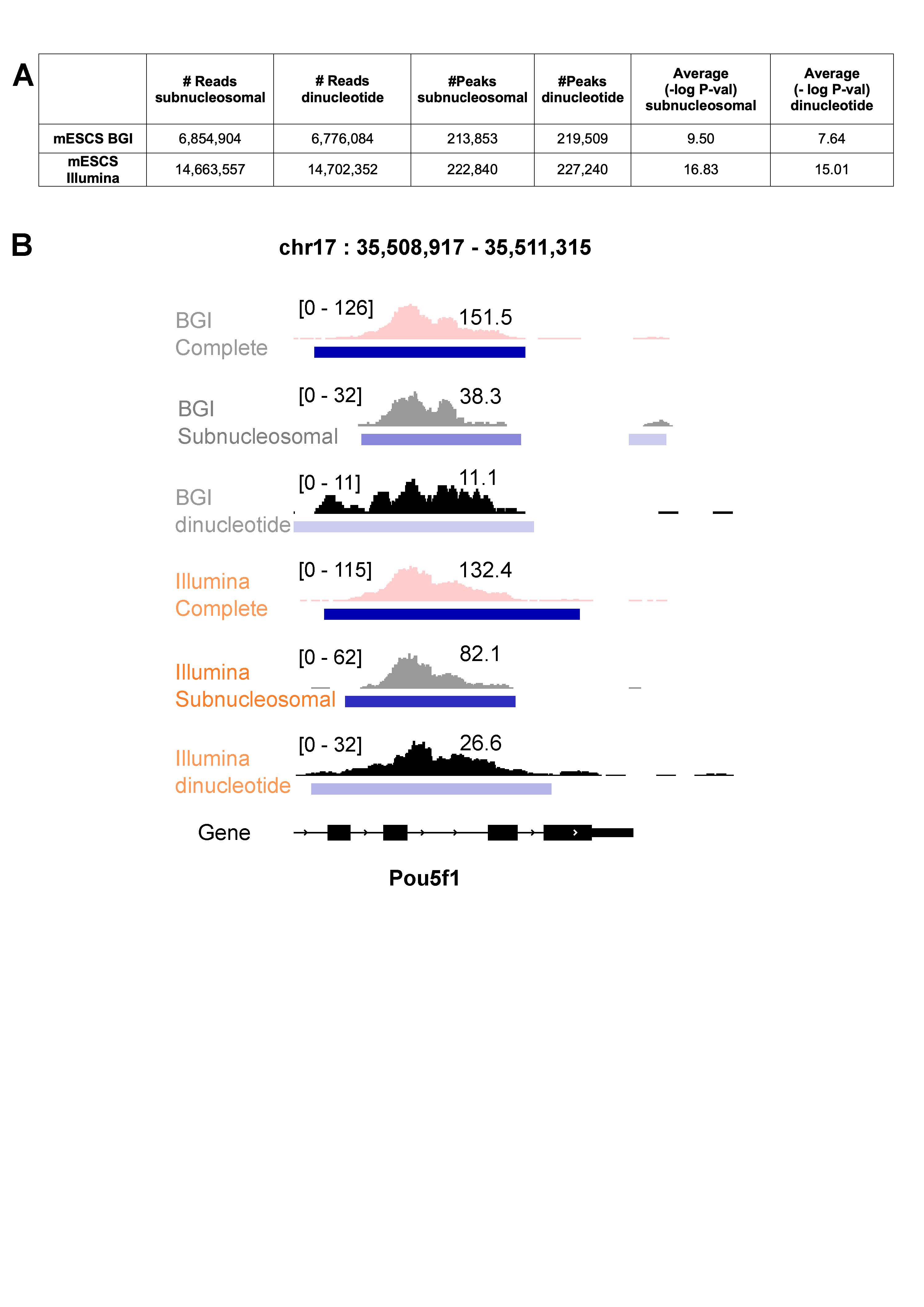

Supplement: Supplementary file 5 [file Image4.JPEG]

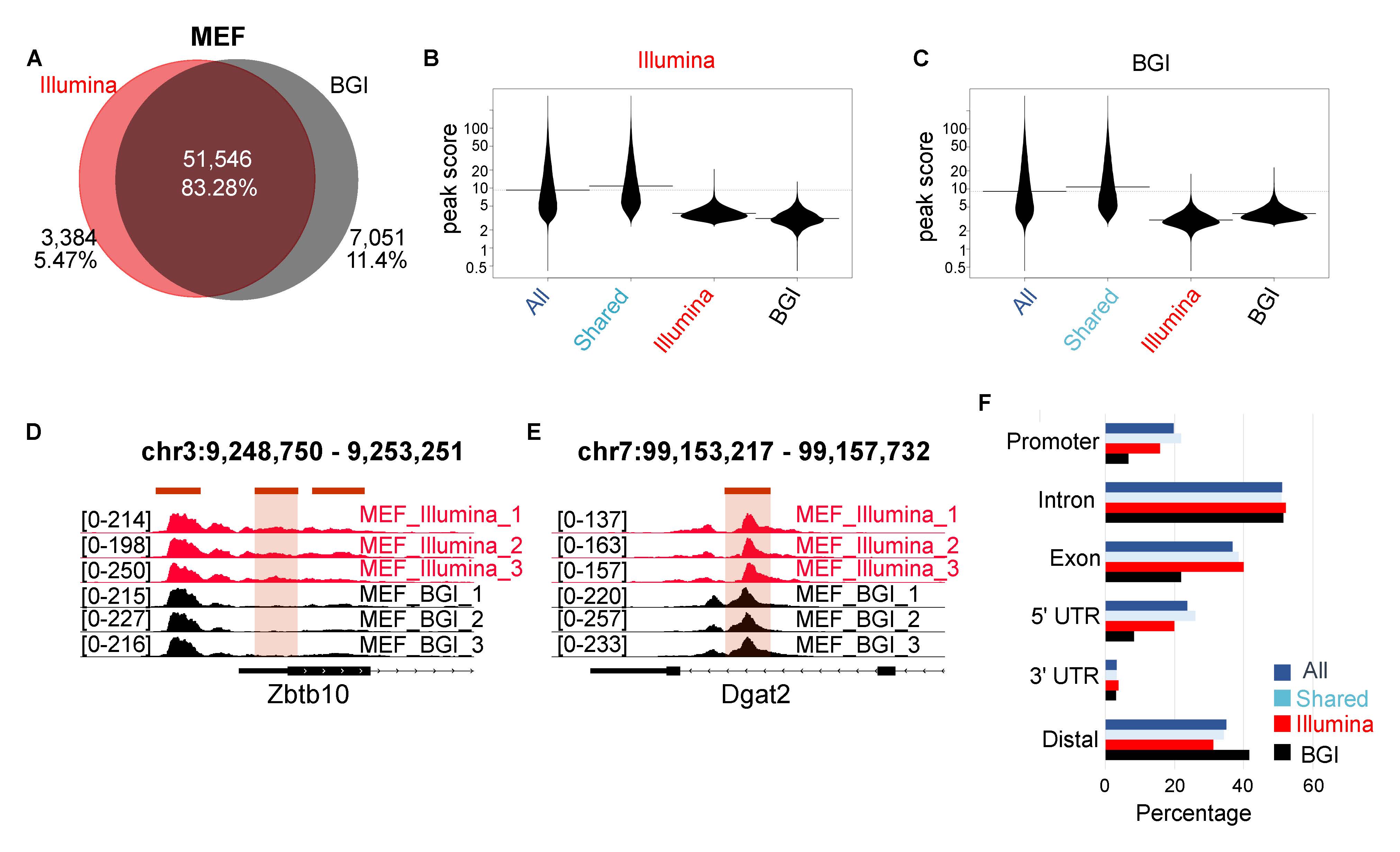

Supplement: Supplementary file 6 [file Image2.JPEG]

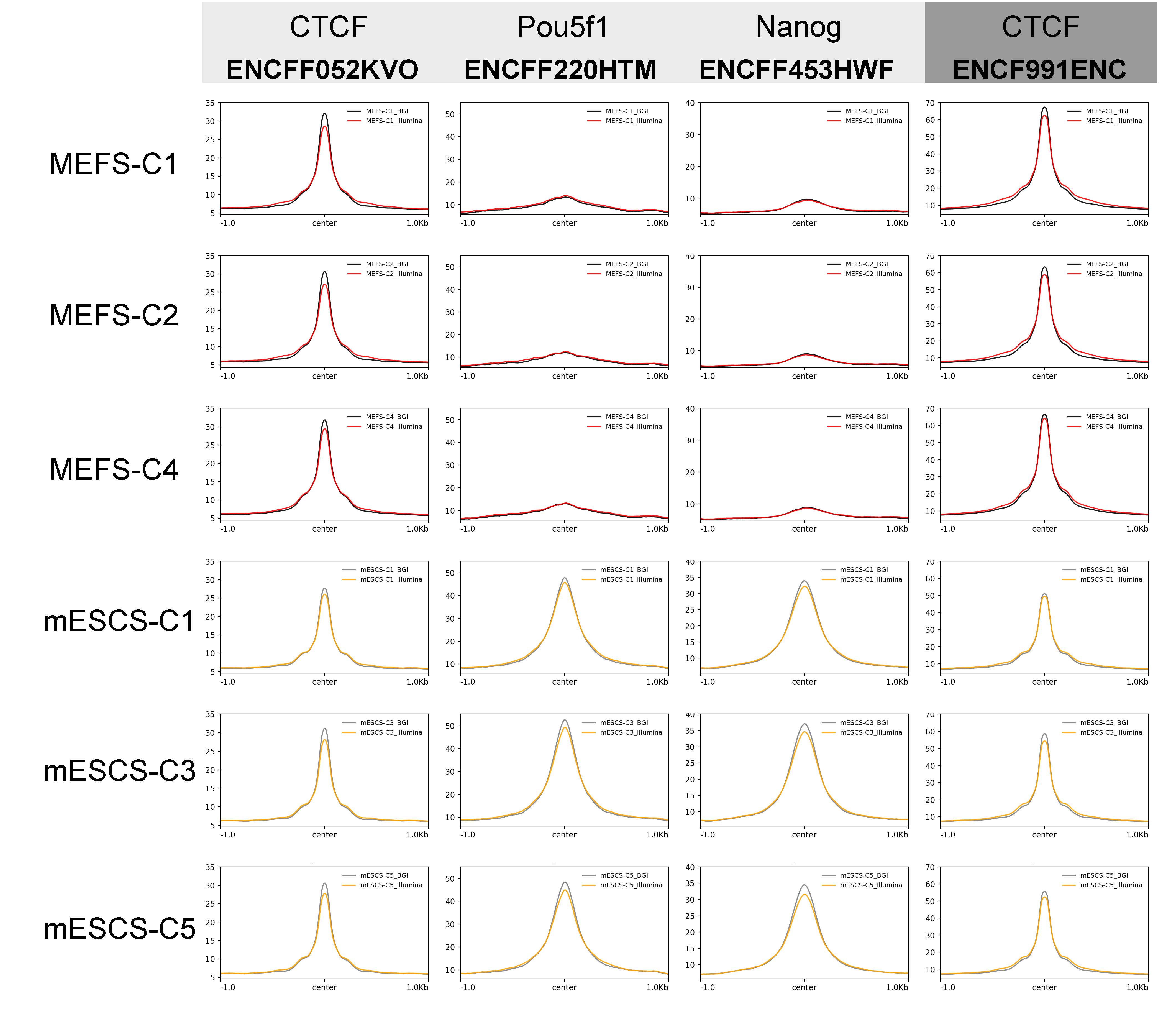

Supplement: Supplementary file 7 [file Image5.JPEG]
